# Commentary: Putting ‘Emotional Intelligences' in Their Place: Introducing the Integrated Model of Affect-Related Individual Differences

**DOI:** 10.3389/fpsyg.2020.00574

**Published:** 2020-04-02

**Authors:** Pablo Ezequiel Flores Kanter, Leonardo Adrián Medrano

**Affiliations:** ^1^Department of Research, Siglo 21 Business University, Córdoba, Argentina; ^2^Department of Psychology, Pontificia Universidad Católica Madre y Maestra, Santiago de los Caballeros, Santiago, Dominican Republic

**Keywords:** emotion, emotion regulation, emotional intellegence, emotional ability, cognitive emotion regulation, stress

Recently, Hughes and Evans (2018) have published an extensive review of variables linked to the concept of emotional intelligence (EI). From a critically developing of the latter construct, they have proposed an integrating model that overcomes the identified limitations. Among these limitations, the faults in content, construct, criteria, and discriminant validity of many of the facets attributed to EI were highlighted. The most substantial criticism was focused on the lack of conceptual delimitation and the absence of an explicit theory linking the traditionally identified facets of EI. The latter is also associated with a lack of integration or distinction with other key variables that have strong prior theoretical and empirical development, such as emotional regulation. In order to resolve these limitations, Hughes and Evans (2018) propose an integrative theoretical model that contemplates the following main variables: (a) ability EI; (b) affect-related personality traits; and (c) emotional regulation. Although we agree with the authors on the advantages of this model and its validity, we consider that the proposed model takes in to account only controlled or elaborative information processing, which generally predominates in benign-safes contexts. In this sense, the model does not contemplate more reactive or automatic affective-cognitive processes, whose predominance is more evident in stressful situations.

In the present brief commentary, the aim is to broaden the Integrated Model of Affect-Related Individual Differences by adding two substantial variables: (a) contextual variables (i.e., stress vs. safety situations); and (b) the particular affective-cognitive responses that can be triggered in each case (i.e., bottom-up vs. top-down). We summarize the revised integrative model in Figure 1. In stress context, the organism responds through a two-way pathway. Path 1 is the first one in temporal terms, triggering the primary negative affective responses. These, in turn, trigger a series of cognitive responses, such as repetitive negative thinking, which have the objective of amplifying the negative affective state. Path 2 is presented in the second instance and entails more controlled, slower, and elaborate cognitive processes, depending mainly on the ability EI. The affective outcome achieved will vary based on the predominance of one or another mode of processing, and the particular relationship that the automatic and elaborative processes maintain with each other (Flores-Kanter et al., 2019; Flores-Kanter, 2020). Although the Figure 1 has heuristic value, it is only illustrative (for an exhaustive development of the neural areas and networks involved in both types of emotional regulation, and functional interactions within and across subcortical and cortical structures, see Park et al., 2019).

**Figure 1:**
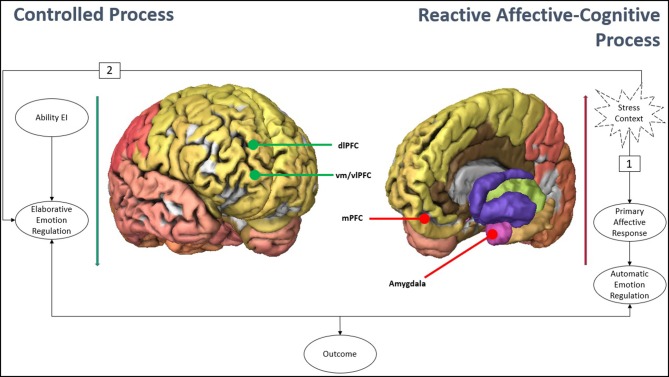
The revised integrated model of affect-related individual differences. Schematic representation of subcortical structures (amygdala) implicated in emotion generation and cortical regions implicated in emotion regulation (both, bottom-up right-brain figure, and top-down left-brain figure). An original figure developed through Brain Explorer software from the Allen Institute for Brain Science (Sunkin et al., 2012).

The revised integrative model is consistent with current developments in affective neuroscience. From these approaches it is proposed, firstly, to differentiate between brain functioning in stress situations and recovery or homeostasis situations (Tobia et al., 2017), and, secondly, that emotion regulation (ER) derived from diverse brain circuits (Hofmann et al., 2012; Beauchaine and Zisner, 2017; Park et al., 2019), involved both in the contextual processing of information (i.e., higher-level cognitive structures) and in the triggering of emotion (i.e., lower level mesolimbic structures). Thus, during an adverse or stressful situation, different and neurodynamically independent brain networks are activated, linked first with negative and positive affect, and, secondly, with processes of regulation of these primary affective responses (McNaughton, 2019). This last functional system of brain networks would not act as an independent factor (unlike affects) but interacts with the systems of negative and positive affect (NA and PA), as a process of regulation and control of affect and behavior (Whittle et al., 2006). About the brain regions involved in emotions and stress response, there would be two different processes that can be activated in the regulation of affection, some ascending and others descending (i.e., bottom-up and top-down emotion regulation; Phillips et al., 2008). The former (bottom-up) are usually called automatic or reactive processes of ER, given that they involve cognitive responses that do not arise from a deliberative process, being automatically induced by the emotional stimulus and involving subcortical structures of generation or affective triggering. On the other hand, top-down processes involve deliberate (effortful and explicit) and reflexive cognitive effort and involve other higher cortex structures. The evidence so far suggests that bottom-up and top-down responses are distinct processes, which comprise the activation of different emotion regulation strategies and maintain a bidirectional relationship with each other (Park et al., 2019).

The differentiation between a more automatic system and a more elaborate one is in line with some psychological theoretical models that recognize the existence of automatic and controlled processes, although with varying denominations (e.g., Kahneman, 2011). Among the different models, dual processing should be highlighted (Beck and Clark, 1997). According to the present comment, the dual-processing model proposes that information processing takes place via a double path. This model has also found support in evidence from the neurosciences. Hofmann et al. (2012) provide a review of the neurobiological mechanisms underlying the cognitive biases and dysfunctional beliefs characteristic of anxiety. The model also finds support in neurobiology. For example, Phelps and LeDoux (2005) have highlighted the primary role of the amygdala in the emotion process and the importance of contemplating an initial and automatic evaluative stage when considering emotional responses.

In conclusion, this Revisited Integrated Model of Affect-Related Individual Differences will make possible a more complex approach. First, the revised model allows contemplating different neurofunctional processes that can be involved in cognitive-affective regulation. Second, the model suggests a more precise explanation of the link that these processes of ER maintain both, with mechanisms of emotional and motivational generation, as well as with mechanisms of self-regulation and affective control, depending on the nature of the particular situation (stressful or benign-safe context).

## Author Contributions

All authors listed have made a substantial, direct and intellectual contribution to the work, and approved it for publication.

### Conflict of Interest

The authors declare that the research was conducted in the absence of any commercial or financial relationships that could be construed as a potential conflict of interest.
